# Multicriteria Decision Making in Supply Chain Management Using FMEA and Hybrid AHP-PROMETHEE Algorithms

**DOI:** 10.3390/s23084041

**Published:** 2023-04-17

**Authors:** Bandar Altubaishe, Salil Desai

**Affiliations:** 1 Department of Supply Chain Management, University of Business and Technology, University of Business and Technology St, Jeddah 23847, Saudi Arabia; b.altubaishe@ubt.edu.sa; 2 Department of Industrial and Systems Engineering, North Carolina Agricultural and Technical State University, Greensboro, NC 27411, USA; 3 Center of Excellence in Product Design and Advanced Manufacturing, North Carolina Agricultural and Technical State University, Greensboro, NC 27411, USA

**Keywords:** analytical hierarchy process (AHP), failure mode and effects analysis (FMEA), internet of things (IoT) sensors, preference ranking organization method for enrichment evaluation (PROMETHEE), supply chain risk management

## Abstract

In today’s global environment, supplier selection is one of the critical strategic decisions made by supply chain management. The supplier selection process involves the evaluation of suppliers based on several criteria, including their core capabilities, price offerings, lead times, geographical proximity, data collection sensor networks, and associated risks. The ubiquitous presence of internet of things (IoT) sensors at different levels of supply chains can result in risks that cascade to the upstream end of the supply chain, making it imperative to implement a systematic supplier selection methodology. This research proposes a combinatorial approach for risk assessment in supplier selection using the failure mode effect analysis (FMEA) with hybrid analytic hierarchy process (AHP) and the preference ranking organization method for enrichment evaluation (PROMETHEE). The FMEA is used to identify the failure modes based on a set of supplier criteria. The AHP is implemented to determine the global weights for each criterion, and PROMETHEE is used to prioritize the optimal supplier based on the lowest supply chain risk. The integration of multicriteria decision making (MCDM) methods overcomes the shortcomings of the traditional FMEA and enhances the precision of prioritizing the risk priority numbers (RPN). A case study is presented to validate the combinatorial model. The outcomes indicate that suppliers were evaluated more effectively based on company chosen criteria to select a low-risk supplier over the traditional FMEA approach. This research establishes a foundation for the application of multicriteria decision-making methodology for unbiased prioritization of critical supplier selection criteria and evaluation of different supply chain suppliers.

## 1. Introduction

With the emergence of new challenges, such as trade wars [1], infectious diseases [2], global warming [3], trade protectionism [4], and rapid technology developments in automation, artificial intelligence [5,6,7,8], internet of things (IoT) sensor [9] virtual reality [10], additive manufacturing [11,12,13,14,15,16], big data [17], and blockchain technology [18], supply chains are evolving to a new dimension. In this context, supplier selection is considered one of the most important strategic decisions to be made by supply chain management (SCM). Manufacturing firms in the U.S. expend substantial financial resources on goods and services. For example, Toyota’s annual purchasing of parts, materials, goods, and services from North American suppliers totaled nearly $32 billion [19]. This significant purchasing expenditure makes a company’s success depend on its right selection of suppliers. By selecting the right supplier, companies can save material costs, increase profits, gain a competitive advantage, and reduce disruption risks. On the other hand, selecting the wrong supplier can result in a variety of problems, such as long lead–times, stock–outs, and the inability to meet customer demand, thereby incurring additional costs [20]. These problems can negatively impact the financial performance of a firm. The COVID–19 pandemic has unprecedentedly disrupted the supply chains (SCs) across the world and has provided an opportunity to test the resilience of both traditional and green suppliers. The flow of both essential and non–essential goods and services has been severely affected [21]. Therefore, understanding the basic requirements for suppliers and having a meaningful evaluation criteria system for optimal supplier selection is paramount. 

The Internet of Things (IoT) technology is the fourth industrial revolution, and the application of IoT sensors across industry is having a significant impact on value creation and revenue streams [22]. An IoT-enabled supply chain is visualized as an intelligent interconnected sensor network that connects multiple tiers of suppliers, contract manufacturers, service providers, distributors, and customers, physically located in different regions of the world [23]. Businesses are using IoT sensors by giving identification tags to their goods/things and connecting relevant information/data from these things to the cloud over the internet [24]. Centrally uploaded data becomes readily available worldwide, and their associated organizations/suppliers/vendors can be contacted for purchase. The IoT sensors and network integration can provide seamless linking between vendors by amassing real-time data, as well as by augmenting visibility of raw materials, finished good, and, finally, by providing real-time quality control on logistics to maximize revenue streams [25]. Thus, IoT–enabled sensor systems drive cost containment, as well as improve time-to-market capabilities in today’s business environment. In essence, the availability of IoT-enabled real-time data can allow stakeholders to make informed decisions at both operational and strategic levels. 

However, most supply chain managers agree that there is no one best way to evaluate and select suppliers with the copious amounts of data being collected across different supply chain tiers by IoT sensors [26]. Dickson [27], for instance, identified 23 different criteria for selecting suppliers, including quality, delivery, performance history, warranties, price, technical capability, and financial position. However, he indicated that quality and delivery are more important than the rest of the 21 criteria. On the other hand, Ellram [28] distinguished between the criteria used for a partnership type of relationship with potential suppliers and the criteria used for traditional buyer–supplier relationships. Weber et al. [29] reviewed 74 different articles to classify criteria and analytical methods for vendor selection into three categories: linear weighting methods, mathematical programming models, and statistical approaches. Boran et al. [30] identified four stages for supplier selection, including (1) definition of the problem, (2) formulation of criteria, (3) qualification, and (4) final selection, respectively. Regardless of the method utilized to evaluate and select a supplier, the goal of the selection process is to reduce purchasing risk and maximize overall value to the company [26]. The vendor with the IoT-enabled process eliminates visibility gaps and injects enormous flexibility into the supply chain network. This criterion is a crucial concern nowadays. Supply chain risk management (SCRM), on the other hand, is one of the key components that contribute to the firm’s success. Supply chain risks can be reduced to a great extent by implementing IoT sensors across multiple tiers and assessing the risks associated with the supplier selection process [31]. Therefore, this research aims to model and optimize the risk assessment in supplier selection. 

There are several techniques available for risk assessment. In our research, we utilized the failure mode and effects analysis (FMEA). FMEA is a widely used engineering analysis technique for preventing errors. FMEA also is performed to identify, prioritize, and eliminate known and potential failures, problems and errors in systems, and products or processes before they reach customers [32]. Moreover, FMEA is a structured technique that can help in identifying all failure modes within a system. The traditional FMEA is solely based on the risk priority number (RPN). The RPN prioritizes the identified potential failure modes. The RPN is computed by multiplying the scores of risk factors. These factors include severity (S), occurrence (O), and detection (D). FMEA has been utilized in a wide range of fields, such as aerospace, chemical, military, automobile, electrical, mechanical, and semiconductor industries [32]. Although this technique has been widely used across different industries, it has limitations. Chang et al. [33] and Liu et al. [34] introduced five issues encountered with RPN in the FMEA method. A key limitation is that different combinations of risk factors may produce the same value of RPN. For example, two different failure modes with values of 4, 3, and 1 and 6, 1, and 2 for (S), (O), and (D), respectively, will have the same RPN as 12. This can result in ambiguity to prioritize different failure modes in supplier selection. Most managers are concerned about the inconsistencies in the ranking of severity, occurrence, and detection attributes and thus may defer FMEA utilization as an assessment tool in a supply chain. Managers are seeking a systematic procedure for rectifying these problems in the FMEA method to integrate the FMEA process into supply chain risk management [34]. In order to overcome the limitations inherent in the traditional FMEA, this research proposes an integration of multi–criteria decision–making (MCDM) methods with FMEA. 

The supplier selection process involves evaluating a large number of suppliers with different capabilities, inherent limitations, geographical locations, intelligent sensor networks, modes of communication, and associated risks. In addition, this selection process generally relies significantly on subjective judgment. Since the supplier selection process involves the evaluation of different criteria and supplier attributes, it can be considered as a multiple criteria decision-making (MCDM) problem [35]. According to Heller [36], using MCDM in risk assessment has proved its effectiveness and reported many advantages, such as supporting the compression of alternatives by using decision matrices and developing a structured method to rank the alternatives. Among different types of MCDM approaches, the analytic hierarchy process (AHP) and the preference ranking organization method for enrichment evaluation (PROMETHEE) are used to enhance the precision of prioritizing failure modes. AHP is used to determine the global weights for each criterion, and the PROMETHEE is implemented to rank suppliers based on the highest net outranking value (lowest supply chain risk). This research establishes a foundation for the application of a multi-criteria decision-making methodology for unbiased prioritization of critical supplier selection criteria and evaluation of different supply chain suppliers. In addition, it provides a supply chain risk assessment tool with high flexibility for supplier selection.

## 2. The Literature Review

The supplier selection process is considered as one of the most important responsibilities of supply chain management within a corporation, especially with the introduction of IoT-based sensing systems [37]. This process has been subject to widespread conceptual and empirical studies in the business management literature [38]. Generally, when a supplier selection decision needs to be made, supply chain management focuses on two aspects. The first aspect focuses on identifying key criteria to be used to evaluate potential suppliers. The second aspect involves determining the evaluation methods to be used to compare different suppliers [39]. The criteria considered in supplier evaluation are industry-specific [40]. In other words, the selection of criteria can be adapted based on the requirements of each industry with data acquisition and augmentation using edge-based sensors. Thus, the following literature review focuses on different methods used to resolve the supplier selection problem.

### Supplier Selection Method

Several studies have been conducted for supplier selection using multicriteria decision-making (MCDM) [41]. In addition, supplier selection problems have been approached by other techniques, including the analytic hierarchy process (AHP), analytic network process (ANP), data envelopment analysis (DEA), fuzzy sets theory (FST), genetic algorithm (GA), goal programming (GP), and simple multi-attribute rating technique (SMART) [42]. However, few researchers have proposed the integration of FMEA with MCDM methods to solve supplier selection problems as a risk mitigation strategy. 

Braglia [43] proposed a new method based on AHP. This method is a multi-attribute failure mode analysis (MAFMA). In this work, FMEA risk factors, such as severity, occurrence, detectability, and expected cost, are considered as decision attributes. Additionally, the causes of failure are considered as alternatives, and the selection of the causes of failure are considered the main objective. Both attributes and alternatives constitute a hierarchical structure of three levels. A comparison matrix was used to calculate the weight of these attributes and the priorities of the causes based on the respective expected cost. In addition, Braglia et al. [44] introduced a new method to estimate the risk priority number (RPN) based on the fuzzy approach of the technique for order preference by similarity to ideal solution (TOPSIS). In Braglia et al.’s [44] study, TOPSIS was used because it allows measurement of the Euclidean distance of an alternative from an ideal objective to improve the risk assessment processes in traditional FMEA. Moreover, a fuzzy logic of TOPSIS was developed to directly evaluate the crisp linguistic assessment of FMEA risk factors (O, S, D) by eliminating the possible errors and uncertainty. 

Chin et al. [45] suggested a new FMEA technique using a group-based evidential reasoning approach in order to capture the diversity, incompleteness, and uncertainty of information provided by FMEA team members. This technique allows FMEA team members to assess risk factors independently and present their ideas individually. This method aggregates risk factors using belief structure at individual levels to generate risk scores in a comprehensive manner. Chamodrakas et al. [46] used a fuzzy analytic hierarchy process (F-AHP) approach for supplier selection in electronic marketplaces. In this study, enforcement of hard constraints on the selection criteria and an application of a modified F-AHP performed on supplier selection were introduced in order to reduce computational complexity. In addition, the use of the F-AHP method facilitates an easier elicitation of user preferences through the reduction of necessary user input.

Yang et al. [47] introduced a modified evidence theory that deals with the multiple evaluations of the risk conducted by experts. This technique deals with different opinions of the FEMA team and multiple failure modes. It also provides simplified discernment frames according to practical engineering applications. The risk factors were fused together as discrete random variables, wherein the RPN was a function of their probabilities. The failure modes were prioritized for their risks based on the mean value of the RPN. This proposed technique was applied to aircraft turbine rotor blades, in which the information of eight failures was evaluated by three different expertises and aggregated together. The result indicated that the risk ranking of the failure modes was consistent with the practical engineering background.

Shaw et al. (2012) used a combination of fuzzy AHP and fuzzy objective linear programming to select the best supplier in order to develop a low-carbon supply chain. The top criteria included in Shaw et al.’s study were cost, demand, gas emission, quality, rejection percentage, the percentage of late delivery, and the greenhouse effect. F–AHP was used to determine the weights of predetermined criteria, and fuzzy objective linear programming was used to select the best supplier.

Chen and Wu [48] proposed a modified failure mode and effects analysis method for selection problems in the supply chain risk environment (MFMEA). They applied the AHP method to determine the weight of each criterion and sub-criterion for supplier selection. FMEA was used to determine the risk priority number of each failure mode to determine the risk priority for an IC assembly company. The result indicated that a corporation could categorize its suppliers more effectively and select a low-risk supply chain partner.

While several studies have been conducted for supplier selection with different approaches that combine decision support methods, limited research has been carried out for supplier risk assessment that combines the MCDM with the FMEA method. Our research proposes a combinatorial approach for risk assessment in supplier selection using the FMEA and the hybrid AHP–PROMOTHEE algorithm. This methodology aims to enhance the precision of the FMEA method, thereby eliminating limitations of the traditional FMEA for choosing optimal criteria and suppliers. 

Modern products often incorporate critical components or sophisticated materials that require specialized technical skills and associated core competencies. Original equipment manufacturers (OEMs) typically resort to vertical integration by outsourcing component assemblies to Tier 2 and Tier 3 suppliers. Thus, suppliers play an important role at every stage of the product lifecycle, ranging from sourcing raw materials and ramping up production to order fulfillment. In the contemporary world, the supply chain economy has become more vulnerable to wars, diseases, and natural disasters. Recent events, such as the COVID–19 pandemic, have caught companies off-guard and resulted in a constant state of disruption, wherein companies are looking for reliable supplier alternatives [49]. Similarly, the Ukraine war [50] and the US–China trade war [51] have forced companies to reevaluate their existing suppliers and add more suppliers to their existing pool. In addition, the industry 4.0 revolution has shifted corporate focus on aligning with digitally vetted suppliers beyond the conventional supplier base [52]. The above–stated supply chain dynamics have resulted in a renewed interest in evaluating suppliers on a relatively frequent interval as compared to the traditional supplier evaluation outlook, which occurred over years. Moreover, the presence of IoT sensors in the supply chains and associated data collection tools can automate information gathering and analysis of new and existing suppliers [53]. Our proposed supplier evaluation strategy can address the above-said issues for both new and existing suppliers.

## 3. Methodology

Though attempts have been made to eliminate the shortcomings of the traditional FMEA process, further enhancements need to be investigated. In this research, a proposed hybrid AHP-PROMOTHEE-based FMEA [54] method is validated on a case study for automotive products [39]. Three criteria were considered, which include: cost, quality, and deliverability based on an IoT sensor network, and nine sub–criteria were employed for supplier selection problems. These criteria were selected based on an exhaustive survey of automotive OEMs and multi-tiered suppliers from the European, American, and Asian auto manufacturers [55].

**Figure 1:**
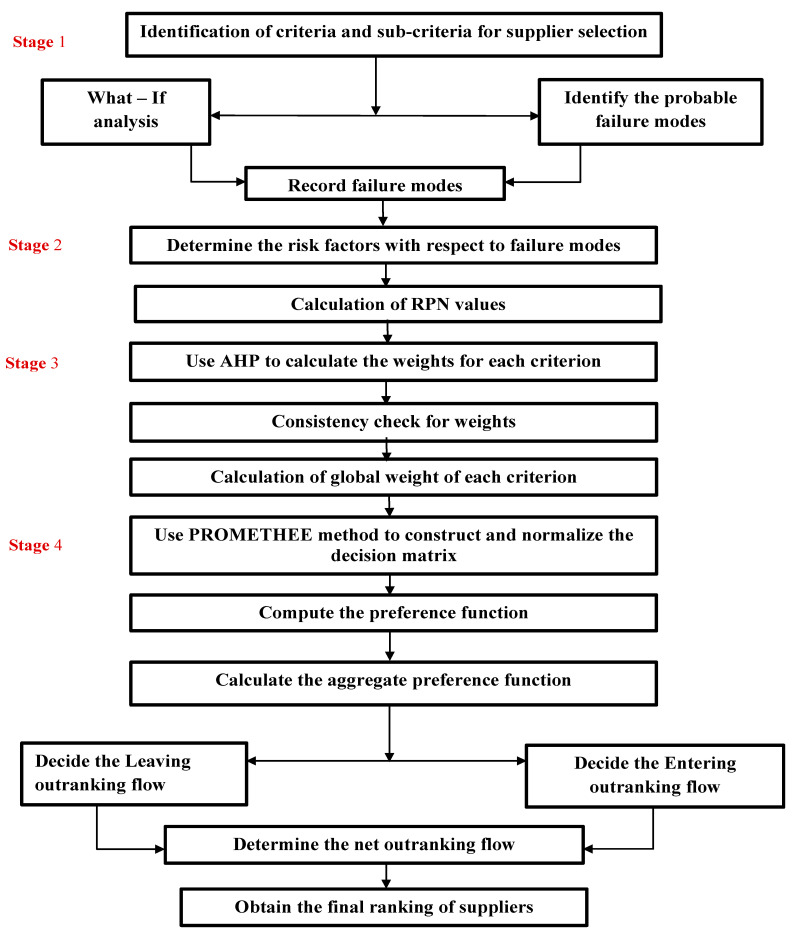
Framework for the combinatorial FMEA and hybrid AHP-PROMETHEE methodology.

The authors used the new failure mode and effects analysis (NFMEA) method, which integrates the fuzzy analytic hierarchy process and FMEA approach. In order to illustrate our methodology, three criteria and nine sub-criteria were selected. Three potential suppliers (A, B, and C) were considered with varying levels of attributes. The proposed combinatorial model integrates FMEA with the AHP–PROMOTHEE approach and consists of four stages for prioritization of the risks identified in the supplier selection process. Figure 1 below shows the four-stage framework for risk assessment and the selection of suppliers.

### 3.1. STAGE (I) Selection of Criteria and Sub-Criteria

In this step, the supplier selection team identifies a set of criteria and sub-criteria to evaluate potential suppliers. Each industry sector, as well as corporation, will have a unique set of criteria based on its supply chain sensor network. Based on the tier level in the supply chain, it may be important to include upstream-tier corporations to determine the selection criteria. The criteria and sub-criteria for supplier selection in our research are shown in Table 1. In this paper, we evaluate three suppliers referred to as Supplier A, Supplier B, and Supplier C, respectively, and the supplier deliverability criteria are based on the IoT–enabled system.

### 3.2. Development of FMEA Documents

In this paper, the FMEA document is constructed for each supplier to record the corresponding risk priority number (RPN). The supplier selection team identifies the probable failure modes based on the selection criteria. A failure mode is referred to the deviation of the criterion from the expected level. By focusing on the selection criteria and sub-criteria, the failure modes are further classified through what–if questions. This step defines how the criteria performance deviates to record all failures.

### 3.3. STAGE (II): Determine the Risk Factors with Respect to Failure Modes

Once all possible failure modes are recorded, the supply chain selection team gave their feedback based on the scale rating of failures. The scale rating consists of a severity (S) rating, which is assigned to evaluate the impact of failure mode, an occurrence (O) rating, which is assigned to reflect the frequency of failures, and a detection (D) rating, which is assigned to reflect the difficulty of detecting the cause of a failure mode. Scale ratings of failures for all factors are given below in Table 2, Table 3 and Table 4.

Table 4 below shows the detection scale based on the degree of collaboration and information exchange within the supply chain departments. The implementation of IoT sensors can aid in identifying failures remotely. More collaboration and information exchange increases the probability of detection [56]

#### Calculation of Risk Priority Number (RPN) Values

The RPNs for failure modes are calculated from expert feedback using the equation RPN = S × O × D. Based on the RPN values, the failure modes are prioritized as per the traditional FMEA method. The highest RPN value corresponds to a higher priority and lowers RPN value corresponds to a lower priority. Table 5 below shows the calculated RPN values for all suppliers. 

It is important to note that, in the traditional FMEA, the RPN value of each failure mode is considered to establish priority for decision-making. According to Table 5, it is clear that the RPN value of 6 occurs for several criteria (failure modes) for each supplier. Thus, it is difficult and ambiguous for the decision-maker to determine the priority of failure modes. This is one of the major limitations of the conventional FMEA method. To overcome this limitation, we propose the hybrid AHP-PROMETHEE methodology that augments the traditional FMEA approach. In this new approach, the AHP algorithm is used to determine the global weights for each criterion, and PROMETHEE is used to rank the RPN based on the global weights.

### 3.4. STAGE (III): Use AHP to Calculate the Weights for Each Criterion

The analytic hierarchy process (AHP) has found a widespread application in decision-making problems, which involves criteria at multi-tier levels [57,58,59]. AHP was developed by Saaty in 1980 to assist decision-makers to reach a consensus decision when conflicting objectives exist [60]. The AHP has the ability to organize the basic rationality by breaking down a problem into smaller parts at different hierarchy levels. Further, a simple pairwise comparison of judgments is conducted to develop priorities in each hierarchy level. In the following section, we describe the AHP hierarchy and calculate the pairwise comparison matrix.

#### 3.4.1. Building the AHP Model and Computing the Weights

**Figure 2:**
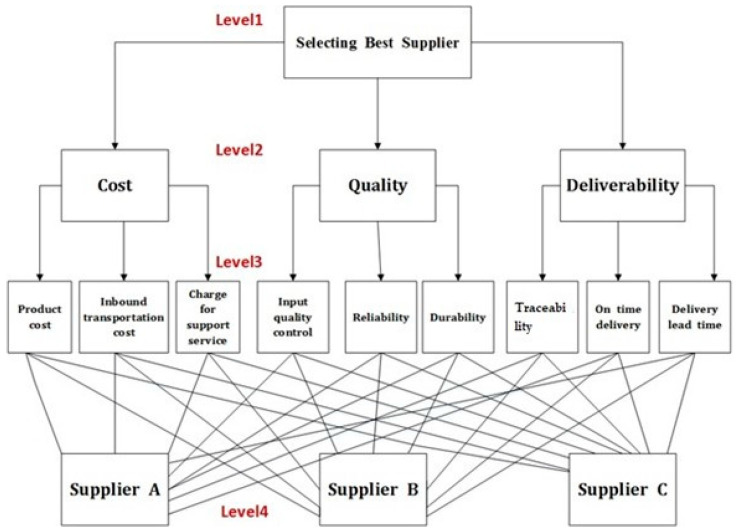
Decision hierarchy for supplier selection.

The AHP model for supplier selection consists of four levels: the goal, the criteria, the sub-criteria, and alternatives. (Figure 2). The first level includes the goal, which is selecting the best supplier. The second level (criteria) contains cost, quality, and deliverability. The third level of the hierarchy consists of nine sub–criteria: product cost, inbound transportation cost, the charge of support service, input quality control, reliability, durability, traceability, on–time delivery, and delivery lead time. The last level of the hierarchy consists of alternatives, which are the different suppliers to be evaluated.

To evaluate the AHP model, the priority weight of each criterion at each level should be calculated. The following steps are implemented to calculate the weight of each criterion: perform a pair–wise comparison matrix. The pair-wise comparison matrix (X_ij_) is based on the expert’s estimation of the relative importance between each criterion. The supply chain team assigns preferences for pairwise comparisons based on the Saaty preference scale values shown in Table 6. 

The preferences of each decision maker are averaged if there is more than one supplier. The pair-wise comparison matrix of the main criteria and sub-criteria based on estimations of the supply chain team is represented in Table 7.

In Table 7 above, each criterion is compared against the other, with the diagonals representing self-comparison of the criteria. For example, the cost criterion in the main criteria matrix is slightly preferred (2) to quality and very strongly preferred (7) to deliverability. Similarly, the criterion of quality is extremely preferred (9) to deliverability. The decision maker (supply chain team) needs to populate the upper half of the matrix, and the lower half is the reciprocal of the upper half. The pair-wise comparison matrices of the sub-criteria with respect to cost, quality, and deliverability are calculated similarly to the main criteria matrix.

A vector of priorities or weighing of criteria and sub-criteria in the matrix are calculated by Equation (1) shown below, where, X_ij_ refers to the importance of criterion i with respect to criterion j, and n refers to the number of criteria.

(1) $$\mathrm{Wi}\;=\;\frac{{\left({⨅}_{j=1}^{n}X_{ij}\right)}^{1/n}}{\sum_{i=1}^{n}{\left({⨅}_{j=1}^{n}X_{ij}\right)}^{1/n}}\;Ɐi=1,2,\ldots .,n$$

#### 3.4.2. Consistency Check for Each Matrix

The consistency check is one of the important features of the AHP method. Consistency check tests the consistency ratio (CR) in each pairwise matrix. CR aims to eliminate any inconsistencies that may occur when assigning the criteria weights due to errors in judgment of the decision makers. A C.R. ≤ 0.10 is considered acceptable, and a perfect consistent ratio is zero. However, if C.R.>0.1, serious inconsistencies may exist, and the pairwise matrix needs to be updated to meet the minimum threshold for further analysis. CRs were obtained for the criteria pairwise matrix and for each sub-criterion by using Equations (2)–(5), shown below, respectively. A consistent matrix (λ_max_) is the largest eigenvalue of a reciprocal matrix of order n. λ_max_ is calculated by Equations (2) and (3).

(2) $$V_{i}=\frac{\sum_{j=1}^{n}W_{j}X_{ij}}{W_{i}}\;{Ɐ}_{i}=1,2,\ldots ..,n$$

(3) $$\lambda \max=\frac{\sum_{i=1}^{n}V_{i}}{n}$$

Consistency index (CI) is calculated by Equation (4).

(4) $$C.I.=\frac{\lambda_{\max}-n}{n-1}$$

CR = CI/RI
(5)

RI stands for random index. RI is based on the number of criteria (n_p_) in each pairwise comparison, as shown in Table 8. In this paper, n_p_= 3 for all levels of hierarchy. 

#### 3.4.3. Calculation of Global Weight

The global weights are calculated by multiplying the weights of the main criteria with the weights of its sub-criteria. The global weight will be used at the end of the prioritization process to obtain the aggregate preference function.

### 3.5. STAGE (IV): Using PROMETHEE Method

The preference ranking organization method for enrichment evaluation (PROMETHEE) method is one of the MCDM methods developed by Brans [61]. PROMETHEE is an outranking method for alternatives while considering several criteria, which may be conflicting [54]. The PROMETHEE family of outranking methods include the PROMETHEE I for partial ranking of the alternatives, as well as the PROMETHEE II for the complete ranking of the alternatives. In this research, we implement the PROMETHEE II method, which is intended to provide a complete ranking of a finite set of feasible alternatives from the best to the worst.

**Figure 3:**
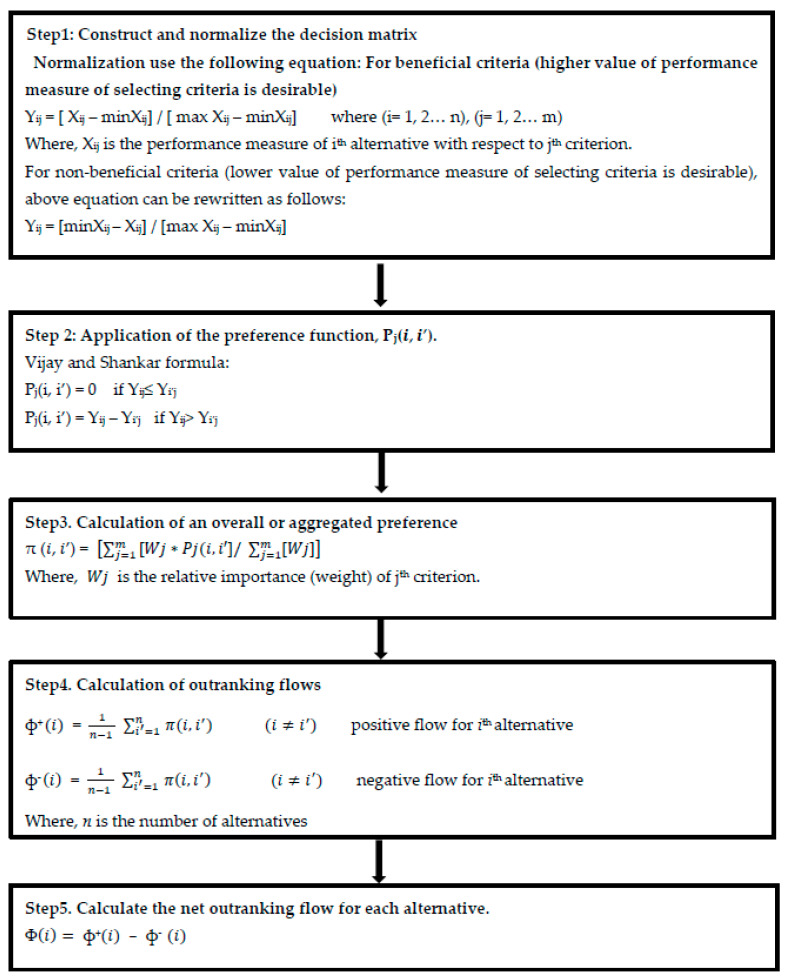
Stepwise procedure for PROMETHEE [62,63].

A stepwise procedure for implementing PROMETHEE is presented in Figure 3 below. The first step involves constructing a normalized matrix by taking into consideration the beneficial and non–beneficial criteria. In step 2, the relevant preference function is applied to each criterion. Further, the aggregated preference index is calculated considering the global weights (Step 3). In step 4, the positive and negative outranking flows for each (i^th^) alternative are calculated. The procedure is completed with the calculation of net outranking flow for each alternative. The ranking of the alternatives depends on the values of ф (i). The alternative with the highest value of ф (i) is the most preferred and vice versa. 

## 4. Results

Our proposed combinatorial risk assessment method using FMEA and hybrid AHP-PROMOTHEE is applied to a case study, as discussed in the methodology section. Using the pairwise comparison matrices, priority weights are calculated for both the main criteria and sub-criteria using Equation (1). Table 9 shows the computation of priority weights for the main criteria and sub-criteria. The supply chain team gives more priority weight to cost (55.5%), followed by quality (38.5%) and deliverability (6%). 

The consistency ratios (C.R.) for all pairwise comparisons among the criteria and sub-criteria above are calculated using Equations (2)–(5). The results of C.R. indicated that all pairwise matrices are valid with a consistency ratio < 0.1, as shown in Table 10.

Before using the PROMETHEE method to rank the supply chain risks, PROMETHEE assumes that the decision-maker is able to weigh the criteria appropriately. Therefore, the global weights are calculated by multiplying the weight of the main criteria with its respective sub-criteria weights. Table 11 shows the global weights. In this case study, the product cost has the highest weight (39.8%) followed by reliability (22.3%), durability (14.2%), and inbound transportation cost (10.6%), respectively.

Assigning priority to each failure mode is a non-trivial problem, which can be addressed by the preference ranking organization method for enrichment evaluation (PROMETHEE) method. The decision matrix for all suppliers (A, B, C) is shown in Table 12. This matrix was populated from the RPN values in Table 5.

The decision matrix is normalized by using the appropriate normalization formula in step 1 from Figure 3. Table 13 shows the normalized decision matrix using the non-beneficial criteria, wherein the lower value of the performance measure is desirable. In our case, the objective is to select a supplier with the lowest supply chain risk, thus the non-beneficial criteria are considered for normalization. 

A paired comparison (Step 2, Figure 3) was conducted for all the supplier pairs using the normalized decision matrix, and the preference function was calculated using the Vijay and Shankar formulas, as shown in Table 14 below.

The aggregated preference function for suppliers was calculated by using the appropriate formula (Step 3, Figure 3). Table 15 shows the aggregated preference matrix, which takes into account the global weights (Table 11).

In the final stage, the entering and leaving flow values were calculated using the appropriate formula (Step 4, Figure 3). Further, the net outranking flow for each alternative was computed (Step 5, Figure 3). The outranking flows for each supplier are based on attaining the lowest supply chain risk. In PROMOTHEE, the higher value of the net outranking corresponds to a preferred alternative. Thus, the best supplier is the one having the highest net outranking value. Table 16 shows the entering flow, leaving flow, and net outranking flow values. 

## 5. Discussion

### 5.1. Contrasting Supplier Selection Methodologies

A comparative analysis was performed to evaluate the supplier selection methodologies. Table 5 shows the risk priority numbers for each sub-criterion for all suppliers calculated using the traditional FMEA model as [31]. As can be seen in Table 5, supplier B has the lowest total (40) and average (6.7) for the RPN among all suppliers. Thus, based on the traditional FMEA model, supplier B is the preferred choice of supplier for the corporation due to its lowest risk potential. In this case study, the failure modes with RPN equal or greater than 6 need improvement. For supplier B, the sub-criteria input quality control, durability, and on-time delivery have a lower RPN as compared to other suppliers. However, the traditional FMEA method has ambiguity when deciding which sub-criteria to improve upon based on a threshold RPN (6), as multiple sub-criteria can have the same RPN. This exacerbates the decision-making of the supply chain team to determine the priorities among sub-criteria for further improvement. This is one of the critical limitations of traditional FMEA, which is addressed in our new approach.

Our combinatorial methodology of FMEA and hybrid AHP-PROMETHEE is a vital tool in solving the supplier selection problem. The proposed methodology allows decision-makers to rank the supplier alternatives effectively and provide valuable guidance in framing supplier selection strategies. An important aspect of the new methodology is the inclusion of weights for supplier selection criteria based on the company policy and preference. Table 17 below depicts a comparative analysis between the traditional FMEA and the proposed methodology for supplier rankings. In the proposed methodology, supplier A is the most preferred supplier with the highest net outranking value of 0.389 (lowest risk). This outcome is in alignment with the company’s product cost reduction strategy (global weighted preference for product cost ~40%). Supplier B was the preferred choice according to the traditional FMEA model. However, supplier B is weak with respect to the product cost (RPN = 6), which contradicts the corporate cost reduction strategy. The second highest global weight was assigned to reliability (22.3%). Taking this weight into consideration, both suppliers A and B had equal RPN = 6. However, the cumulative contribution of these two highest criteria exceed 60% of the global weights, which drive the strategic decision making of the enterprise. Accordingly, supplier A is in alignment with the company’s priorities, which are further evaluated using the PROMETHEE algorithm. 

We also compared our results with a recent automotive supplier selection methodology, as per the ISO 9000/TS16949 standards [64]. Herein, each supplier was allocated a weighted sum of the three main criteria, and the supplier with the highest performance score was selected and ranked over the others. As can be seen from Table 17, the highest performance score was attributed to supplier B, followed by supplier A and supplier C. Supplier C was rated the lowest and consistent across all methods. It is evident that inconsistencies for supplier B have been rectified in our proposed method. Thus, the incorporation of the hybrid AHP-PROMETHEE enables the corporation to prioritize failure modes based on the global weight of all criteria and rank each supplier based on lowest supply chain disruption.

### 5.2. Implications for Supply Chain Risk Management

The application of the proposed methodology to real-world scenarios could include a large number of criteria and sub-criteria. This would involve evaluating several data sets obtained through IoT sensors and pairwise comparisons constituting big data analytics. The proposed method can be coded into a sub-routine source code, which can conveniently be adopted and implemented by any industry. Each of the sub-systems (FMEA, AHP, and PROMETHEE) has been coded into commercial software packages. However, the integration and customization of these methods, as demonstrated in this paper, would be a valuable asset to supply chain risk management. Moreover, our approach provides flexibility to include user-chosen supplier selection criteria that can be tailored to different industry sectors that are now enabled with multi–tiered IoT sensors. 

### 5.3. IoT Sensors in Supply Chain Risk Management

IoT sensors enable a digitally connected seamless supply chain environment with multiple sensors on edge-networks that acquire, process, and transmit data pertaining to various aspects of SCM [65]. In our case study, these IoT sensors provide useful data metrics to evaluate each supplier criteria based on the industry type. The sensors mentioned in Table 18 provide parameter ranges encountered by materials, devices, and products within a supply chain. These quantifiable data sets assist in making judicious decisions with regards to cost, quality, and deliverability criteria chosen for our case study. However, these parameters and data acquisition sensors can be varied to suit other applications and industry segments.

## 6. Conclusions

With globalization, supply chains have been transformed due to the internet of things (IoT)-based sensors across different tiers. Thus, they have become increasingly vulnerable to cyber disruptions, as organizations lose millions of dollars because of cost instability, supply disruption, and non-compliance fines that negatively impact the organizational brand. Supply chain risk management (SCRM) is one of the key components for an effective supply chain. This research develops a combinatorial approach for risk assessment in supplier selection using FMEA with hybrid AHP-PROMETHEE methods. A four-stage framework is developed and applied to a case study with three suppliers and nine sub-criteria. The supplier selection team identified company-specific criteria to evaluate potential suppliers. FMEA rubrics for severity (S), occurrence (O), and detection (D) ratings were developed. Each supplier was evaluated to determine the risk priority number for the sub-criteria based on expert opinion. A multi-tier hierarchy of criteria and sub-criteria were generated based on the company-specific preferences by the supply chain team. The AHP method was applied for pairwise comparisons on a vector of priorities to generate global weights for the sub-criteria. The consistency check for criteria and sub-criteria matrices indicated an acceptable consistency ratio of less than 0.1. The AHP generated weights were implemented in the PROMETHEE algorithm to rank suppliers based on the lowest risk of supply chain disruption. The proposed method was contrasted against the traditional FMEA method and showed significant improvements while considering company-specific criteria for risk management. Moreover, our approach offers a feedback mechanism for suppliers to improve on their core competencies in order to meet these criteria and minimize their risk to the supply chain. This method provides the decision makers (supply chain team) an efficient tool to rank candidate suppliers based on their risk potential. In addition, it entails guidance for framing supplier selection risk mitigation strategies.

## Figures and Tables

**Table 1:** Criteria and sub-criteria for supplier selection.

Criterion	Sub-Criterion
Cost	Product cost Inbound transportation cost Charge of support service
Quality	Input quality control Reliability Durability
Deliverability	Traceability On–time delivery Delivery lead time

**Table 2:** Severity (S) scales of criteria.

Severity	Rating	Description
**Extreme**	**5**	**Cost:** supplier offers very expensive raw material **Quality:** supplier offers high level of defective material **Deliverability:** supplier always misses on-time delivery
**high**	**4**	**Cost:** supplier offers high-priced raw material **Quality:** supplier offers many defective materials **Deliverability:** supplier misses on-time delivery most of the time
**Moderate**	**3**	**Cost:** supplier offers slightly high-priced raw material **Quality:** supplier offers some defective material **Deliverability:** supplier misses some on-time delivery
**Low**	**2**	**Cost:** supplier offers medium-priced raw material **Quality:** supplier offers small amount of defective material **Deliverability:** supplier rarely misses on-time delivery
**Minor**	**1**	**Cost:** supplier offers cheap raw material **Quality:** supplier offers very few defective material **Deliverability:** supplier very rarely misses on-time delivery

**Table 3:** Occurrence (O) Scales of Criteria.

Occurrence	Rating	Description
**Extreme**	**5**	**Cost:** supplier always offers very expensive raw material **Quality:** supplier always offers high level of defective material **Deliverability:** supplier always misses on-time delivery
**high**	**4**	**Cost:** supplier usually high-priced the raw material **Quality:** supplier often offers many defective materials **Deliverability:** supplier misses on-time delivery most of the time
**Moderate**	**3**	**Cost:** supplier often offers slightly high-priced raw material **Quality:** supplier offers some defective amount of material **Deliverability:** supplier misses some of the on-time delivery
**Low**	**2**	**Cost:** supplier rarely offers expensive raw material **Quality:** supplier rarely offers defective material **Deliverability:** supplier rarely misses the on-time delivery
**Minor**	**1**	**Cost:** supplier very rarely offers expensive raw material **Quality:** supplier very rarely offers defective material **Deliverability:** supplier very rarely misses on-time delivery

**Table 4:** Detection (D) scales of criteria.

Detection	Rating	Description	Probability of Detection (%) for All Criteria
**Remote**	**5**	No/limited collaboration and information exchange	0–5
**Low**	**4**	Low collaboration and information exchange	6–25
**Moderate**	**3**	Moderate collaboration and information exchange	26–50
**High**	**2**	High collaboration and information exchange	51–75
**Very high**	**1**	Very high collaboration and information exchange	76–100

**Table 5:** Calculation of RPN for Supplier A, B, and C.

		Supplier A				Supplier B				Supplier C			
**Criterion**	Sub-criterion	S	O	D	**RPN**	S	O	D	**RPN**	S	O	D	**RPN**
**Cost**	Product cost	2	2	1	**4**	3	2	1	**6**	3	2	1	**6**
	Inbound transportation cost	3	2	1	**6**	2	3	1	**6**	2	3	1	**6**
	Charge of support service	3	2	1	**6**	2	2	1	**4**	2	2	1	**4**
**Quality**	Input quality control	2	3	1	**6**	1	2	1	**2**	2	2	1	**4**
	Reliability	3	2	1	**6**	2	3	1	**6**	3	3	1	**9**
	Durability	2	4	1	**8**	3	1	1	**3**	4	3	1	**12**
**Deliverability**	Traceability	4	2	1	**8**	3	2	1	**6**	3	2	1	**6**
	On time delivery	2	3	1	**6**	1	3	1	**3**	2	1	1	**2**
	Delivery lead time	3	2	1	**6**	2	2	1	**4**	3	3	1	**9**
	**Total**				**56**				**40**				**58**
	**Average**				**9.3**				**6.7**				**9.7**

**Table 6:** Saaty preference scale values for pairwise comparison.

Preference	Rating Score
Extremely Preferred	9
Very, Very Strong	8
Very Strongly Preferred	7
Strong Plus	6
Strongly Preferred	5
Moderate Plus	4
Moderately Preferred	3
Weak or Slight	2
Equally Preferred	1

**Table 7:** Pair-wise comparison matrix of the main criteria and sub-criteria.

**Pair-wise comparison matrix of the main criteria**			
**Criterion**	Cost	Quality	Deliverability
Cost	1	2	7
Quality	1/2	1	9
Deliverability	1/7	1/9	1
**Pair-wise comparison matrix of the sub-criteria with respect to cost**			
**Sub-criterion**	Product cost	Inbound transportation cost	Charge for support service
Product cost	1	5	7
Inbound transportation cost	1/5	1	3
Charge of support service	1/7	1/3	1
**Pair-wise comparison matrix of the sub-criteria with respect to quality**			
**Sub-criterion**	Input quality control	Reliability	Durability
Input quality control	1	1/7	1/7
Reliability	7	1	2
Durability	7	1/2	1
**Pair-wise comparison matrix of the sub-criteria with respect to deliverability**			
**Sub-criterion**	Traceability	On time delivery	Delivery lead time
Traceability	1	1/5	1
On time delivery	5	1	7
Delivery lead time	1	1/7	1

**Table 8:** Random index for number of criteria (n_p_) in pairwise comparison [60].

**n_p_**	1	2	3	4	5	6	7	8	9
**RI**	0	0	**0.52**	0.89	1.11	1.25	1.35	1.4	1.45

**Table 9:** Calculation of priority weights for the main criteria and sub-criteria.

**Priority weights for the main criteria**								
**Criterion**	**Cost**	**Quality**	**Deliverability**	**Cost** **weight**	**Quality weight**	**Deliverability** **weight**	**Total weight**	**Priority** **weight**
**Cost**	1	2	7	0.609	0.643	0.412	1.663	0.555
**Quality**	0.500	1	9	0.304	0.321	0.529	1.155	0.385
**Deliverability**	0.143	0.111	1	0.087	0.036	0.059	0.181	0.060
**Total**	1.643	3.111	17	1	1	1	3	1
**Priority weights with respect to cost**								
**Criterion**	**Product cost**	**Inbound transportion cost**	**Charge of support service**	**Product cost weight**	**Inbound transportation cost weight**	**Charge of support service weight**	**Total weight**	**Priority** **weight**
**Product cost**	1	5	7	0.745	0.789	0.636	2.171	0.724
**Inbound transportation cost**	0.2	1	3	0.149	0.158	0.273	0.580	0.193
**Charge of support**	0.143	0.333	1	0.106	0.053	0.091	0.250	0.083
**Total**	1.343	6.333	11	1	1	1	3	1
**Priority weights with respect to quality**								
**Criterion**	**Input quality control**	**Reliability**	**Durability**	**Input quality control weight**	**Reliability** **weight**	**Durability** **weight**	**Total weight**	**Priority** **weight**
**Input quality control**	1	0.143	0.143	0.067	0.087	0.045	0.199	0.066
**Reliability**	7	1	2	0.467	0.609	0.636	1.712	0.571
**Durability**	7	0.5	1	0.467	0.304	0.318	1.089	0.363
**Total**	15	1.643	3.143	1	1	1	3	1
**Priority weights with respect to deliverability**								
**Criterion**	**Traceability**	**On time delivery**	**Delivery lead time**	**Traceability weight**	**On time** **delivery weight**	**Delivery** **lead time** **weight**	**Total weight**	**Priority** **weight**
**Traceability**	1	0.200	1	0.143	0.149	0.111	0.403	0.134
**On time delivery**	5	1	7	0.714	0.745	0.778	2.237	0.746
**Delivery lead time**	1	0.143	1	0.143	0.106	0.111	0.360	0.120
**Total**	7	1.343	9	1	1	1	3	1

**Table 10:** Summary of C.R results.

Pairwise Comparison	λmax	C.I	R.I for 3	C.R < 0.1
Main criteria	3.10	0.05	0.52	0.097
Sub-criteria with respect to cost	3.07	0.03	0.52	0.06
Sub-criteria with respect to quality	3.05	0.03	0.52	0.05
Sub-criteria with respect to deliverability	3.01	0.01	0.52	0.1

**Table 11:** Global weight of sub-criteria.

Main Criterion	Weight of the Main Criterion (1)	Sub-Criterion	Weight of Sub-Criterion (2)	Global Weight (3) = (1) × (2)
Cost	0.55	Product cost	0.724	**0.398**
		Inbound transportation cost	0.193	**0.106**
		Charge of support service	0.083	**0.046**
Quality	0.39	Input quality control	0.066	**0.026**
		Reliability	0.571	**0.223**
		Durability	0.363	**0142**
Deliverability	0.06	Traceability	0.134	**0.008**
		On–time delivery	0.746	**0.045**
		Delivery lead time	0.120	**0.007**
			**Total Weight**	**1.000**

**Table 12:** Decision matrix for suppliers in the PROMETHEE.

Supplier	Product Cost	Inbound Transportation Cost	Charge of Support Service	Input Quality Control	Reliability	Durability	Traceability	On Time Delivery	Delivery Lead Time
A	4	6	6	6	6	8	8	6	6
B	6	6	4	2	6	3	6	3	4
C	6	6	4	4	9	12	6	2	9

**Table 13:** Normalized decision matrix for supplier selection.

RPN of Supplier	Product Cost	Inbound Transportation Cost	Charge of Support Service	Input Quality Rate	Reliability	Durability	Traceability	On Time Delivery	Delivery Lead Time
A	1	0	0	1	0	0.556	0	1	0.6
B	0	0	1	0	0	0	1	0.25	1
C	0	0	1	0.5	1	1	1	0	0

**Table 14:** Preference functions for all supplier pairs.

RPN of Supplier	Product Cost	Inbound Transportation Cost	Charge of Support Service	Input Quality Rate	Reliability	Durability	Traceability	On Time Delivery	Delivery Lead Time
(A,B)	1	0	0	1	0	0.555556	0	0.75	0
(A,C)	1	0	0	0.5	0	0	0	1	0.6
(B,A)	0	0	1	0	0	0	1	0	0.4
(B,C)	0	0	0	0	0	0	0	0.25	1
(C,A)	0	0	1	0	1	0.444444	1	0	0
(C,B)	0	0	0	0.5	1	1	0	0	0

**Table 15:** Aggregated preference function for suppliers.

RPN of Supplier	A	B	C
A	Nil	0.536024	0.459927
B	0.05676	Nil	0.018391
C	0.339333	0.37706	Nil

**Table 16:** Outranking flows in PROMOTHEE for suppliers.

Supplier	Leaving Flow	Entering Flow	Net Outranking
A	0.543	0.153	0.389
B	0.289	0.205	0.085
C	0.0614	0.536	−0.475

**Table 17:** Benchmarking of comparative supplier rankings methodologies.

Traditional FMEA			ISO 9000/TS16949 Method		Proposed Method	
Supplier	Traditional total RPN	Rank	Performance Score	Rank	Net Outranking	Rank
A	56	2	0.61	2	0.389	1
B	40	1	0.65	1	0.085	2
C	58	3	0.53	3	–0.475	3

**Table 18:** Impact of IoT sensors on supply chain logistics.

IoT Sensor	Sensor Modality	Parameter Tracked	SCM Impact
Thermal Sensors	Thermistor, Infrared, In situ thermocouple	Temperature range and exposure limits	Quality of perishable or temperature sensitive shipment
Moisture sensors	Psychrometer, hair tension	Humidity range and exposure limits	Quality of hygroscopic materials/products
Light sensors	Photoresistor, photodiode	Exposure to UV, Visible and infrared spectrum	Light sensitive materials/products
Acoustic sensors	Hydrophone, geophone	Frequency range and exposure limits	Vibration sensitive devices and products
Pressure and proximity sensors	Doppler radar, occupancy detector	Multidimensional force/torque loading	Load sensitive fragile materials/products
Image sensors	Active pixel sensor, charge–coupled device	Dimensional accuracy, Particle counter	Quality of materials/products manufactured and shipped
Chemical sensors	Electrochemical nose, Impedance array	Trace particulate content (ppm/ppb)	Toxicity and impurities in materials/products
Gyroscope sensors	Accelerometers	Angular velocity gradients	Stability of devices/products in shipment
Motion sensors	Ultrasonic, infrared, radar, LIDAR	GPS coordinates with time stamps	Assembly operations and shipment tracking

## Data Availability

Not applicable.
